# Effectiveness of flipped classroom vs traditional lectures in radiology education: A meta-analysis

**DOI:** 10.1097/MD.0000000000022430

**Published:** 2020-10-02

**Authors:** Lingling Ge, Yuntian Chen, Chunyi Yan, Zhengwen Chen, Jiaming Liu

**Affiliations:** a West China Hospital, Sichuan University, Chengdu, China; b Radiological Department, West China Hospital, Sichuan University, Chengdu, P.R. China; c Department of Urology, Institute of Urology, West China Hospital, Sichuan University, Chengdu, China.

**Keywords:** effectiveness, flipped classroom, meta-analysis, radiology education

## Abstract

**Background:**

With the convert of educational concept, flipped classroom has been adopted gradually in radiology courses as a new teaching mode. Considering no evidence has been concluded to illustrate the effectiveness of of flipped classroom over traditional instructor-centered lectures in radiology education, this meta-analysis was conducted to provide empirical evidence for the reform of pedagogical.

**Methods:**

Studies were retrieved from six databases, including Pubmed, Embase, Web of Science, Wanfang Data, CNKI, and VIP, from their inception to 16 February 2020. Literature selection and data extraction were completed by two reviewers independently. The effect size of each index was expressed as the odds ratio (OR) for a categorical variable and standard mean difference (SMD) for a continuous variable, each with corresponding 95% confidence interval (95% CI).

**Results:**

A total of 19 studies with 2114 participants were deemed to be eligible for inclusion. The results of this meta-analysis indicated that: the newly emerged flipped classroom represented significant advantage versus traditional lecture in improving theoretical performance (SMD 1.12, 95% CI 0.61–1.63, *P* < .001), as well as in cultivating students’ practical skills (SMD 2.59, 95% CI 1.69–3.59, *P* < .001). In the subjective findings of investigation, more positive responses were attained in students who took radiology subjects in flipped classroom, covering course satisfaction (OR 1.70, 95% CI 1.35–2.14, *P* < .001), improvement of teamwork ability (OR 1.80, 95% CI 1.21–2.67, *P* = .004), self-directed learning and reflection (OR 1.98, 95% CI 1.31–2.97, *P* = .001), and subjective cognition on consolidation of knowledge mastery (OR 1.38, 95% CI 1.19–1.60, *P* < .001).

**Conclusion:**

Flipped classroom displays multiple advantages versus traditional lecture-based teaching mode, which is well worth further promoting and applying in the process of radiology education.

## 1 Introduction

As a bridge course connecting basic and clinical disciplines, radiology has been attached great importance in the era witnessing medical and technological breakthroughs. With respect to the necessity of organic combination of image features and clinical manifestations, radiology education pays more attention to the collision of multidisciplinary disclosure and the integration of theory and practice, putting forward higher requirements for medical students’ logical thinking disposition and autonomous learning ability.^[1]^ While in the process of radiology education, the cramming instructor-centered teaching method is still the predominant mode adopted by most institutes, which focuses on the knowledge infusion and academic performance improvement instead of laying emphasis on the cultivation of practical skills. Acting as a passive recipient of information in teacher-oriented pedagogy, students are accustomed to learning by rote without understanding.[^2^,^3^]

Confronting the malpractice that the traditional teaching mode is inadequate in meeting the training standards of medical talents in this period, a paradigm shift emerges with the term “flipped classroom” coined by Jonathan Bergmann and Aaron Sams in 2012. The proliferation of this brand-new pedagogical approach subverts the traditional instructor-centered and lecture-based in-class contents by integrating the concept of blended learning. To make better use of the limited class time and realize the internalization of knowledge, the network platform in combination with prerecorded instructional resources enable students to complete knowledge learning and supplementary expansion in advance, without strict time and place restrictions. In turn, the liberated in-class time can be used for problem solving and group discussion to promote students’ understanding of knowledge points and facilitate the formation of a comprehensive knowledge system,^[4]^ which further prepares medical students to deal with unexpected challenges encountered in the process of disease diagnosis and clinical decision making.^[5]^

Recent years have witnessed the accelerated application of flipped model in various fields of health professions education, such as nursing,[^6^,^7^] pharmacy,[^8^,^9^] and other medical subjects. It widely acknowledged that the model of “flipped classroom” or “inverted classroom” can make up for the shortcomings of the traditional model and produce desire results through stimulating the enthusiasm of active learning.[^10^,^11^] Up to now, there also exist several studies to explore the value of the teaching-learning method evolution in the education of radiology courses. However, no scientific evidence has been concluded to illustrate the objective judgment as well as the subjective evaluation of this innovatively proposed paradigm by flipping the classroom.

Therefore, we conducted this meta-analysis to systematically evaluate the effectiveness of flipped classroom vs traditional lectures, so as to provide evidence-based guidance for the reform of pedagogical approaches.

## 2 Method

### 2.1 Study consideration

This study was carried out under the guidance of the Preferred Reporting Items for Systematic Reviews and Meta-analysis (PRISMA). Considering that the meta-analysis was performed based on observational studies and did not involve patients, no ethical approval was warranted.

### 2.2 Search strategy

Relevant studies were retrieved from 6 main databases, including Pubmed, Embase, Web of Science, the Wanfang Database, the China National Knowledge Infrastructure (CNKI), the Chinese Scientific Journals Database (VIP), from their inception to February 16, 2020, without specific language restriction. To obtain a wider range of potentially eligible literature, the following keywords were selected: “radiolog∗ OR imag∗ OR CT OR MRI OR ultrasound” AND “flip∗ OR invert∗”. The search strategy was imported as a string and searched independently in these 6 databases.

### 2.3 Inclusion and exclusion criteria

#### 2.3.1 Study design

We included studies designed to explore the effectiveness of flipped classroom in radiology education in comparison with traditional didactic or lecture-based pedagogy, with specific indicators focused on students’ objective evaluation or subjective cognition.

#### 2.3.2 Participants

Medical students from the specific education system learning on the same radiology course topics divided into experimental and control group were included in this meta-analysis.

#### 2.3.3 Intervention

Flipped classroom as a new paradigm was adopted in the experimental group, which involves prerecorded online materials, out-class self-directed learning, and in-class problem-based discussion, while traditional teacher-centered teaching method was conducted in the control group as comparison.

#### 2.3.4 Outcome

The main indicators include at least one of the following: theoretical examination performance; practical examination performance; course satisfaction; cooperation ability; self-directed thinking ability; and theoretical knowledge mastery.

#### 2.3.5 Exclusion criteria

Published studies without required control group; without sufficient extractable data or calculable effect size; and review articles.

## 3 Data extraction method

For studies that fulfilled all the inclusion criteria, 2 reviewers independently extracted data involving first author, published year, sample size, radiology course type, medical student level, intervention measures, contrast pedagogy, and outcome indicators. All data were cross-checked after extracting, and disagreements were solved by consensus or jointly discussed by the third reviewer if necessary.

### 3.1 Methodological quality assessment

The Newcastle–Ottawa scale (NOS) was employed to assess the quality of studies in our meta-analysis, due to the inclusion of quite a large proportion of nonrandomized controlled trails. In the process of quality assessment, the following items were taken into consideration: sample size, principle of randomization, implementation of blinding, allocation concealment, control for key factors, and outcome assessment. The total score of quality ranging from 0 to 11, and the studies awarded 5 points or more were regarded as high-quality ones.

### 3.2 Statistical analysis

For qualitative analysis, we used the Stata/SE version 15.1 (StataCorp, College Station, TX). The odds ratio (OR) and standard mean difference (SMD) with corresponding 95% confidential intervals (95% CIs) were adopted to compute the effect size of categorical variables and continuous variables, respectively. The difference was statistically significant when 2-tailed *P* < .05. *I* ^2^ statistics were used for heterogeneity assumption, of which 50% was taken as the cut-off value. If *I* ^2^ < 50%, no significant heterogeneity was seen in all published articles and fixed effect model was then used for analysis. In contrary, random effect model was employed when heterogeneity existed (*I* ^2^ > 50%). If heterogeneity existed among included studies, subgroup analyses were conducted to explore the source.

### 3.3 Publication bias assessment

Publication bias was assessed by Begg and Egger test with metabias user-written package in Stata/SE version 15.1 and displayed in the visual form of funnel plot. Two-tailed *P* < .05 in Egger test was regarded as significant publication bias and proved the evidence of asymmetry.

## 4 Results

### 4.1 Literature search results

**Figure 1:**
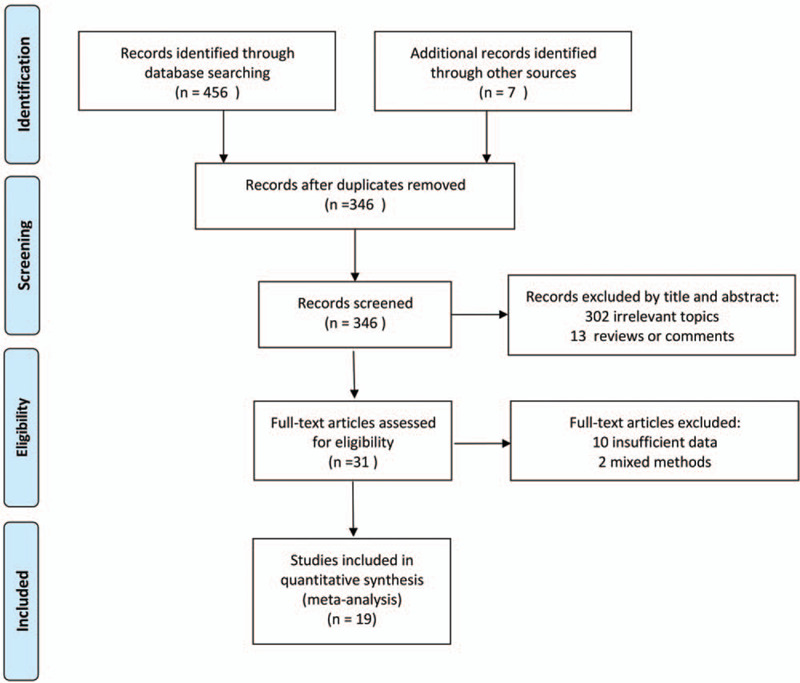
Flow-process diagram for the study selection and inclusion. The flow-process diagram describing the process of literature search according to the PRISMA guidelines, including the reasons for exclusion of studies.

A total of 456 literature search results were initially retrieved from all 6 databases and another 7 were identified through references; altogether 117 of them were removed for duplication. After filtering with titles, abstracts, and subsequent full-text screening, 19 records meeting both eligible characteristics and sufficient data with 2114 participants were eventually included. The flow-process diagram of records selection and inclusion is presented in Figure 1.

### 4.2 Characteristics of included studies

The characteristics of all included studies are summarized in Table 1.[^12^,^13^,^14^,^15^,^16^,^17^,^18^,^19^,^20^,^21^,^22^,^23^,^24^,^25^,^26^,^27^,^28^,^29^,^30^] The publication year of all 19 articles covering a period between 2015 and 2019 with sample sizes ranging from 40 to 240 medical students in participation. A total of 9 nonrandomized trials (NRCTs) and 10 randomized trials (RCTs) were included into our meta-analysis. The studies examined different pedagogical approaches in various radiology subjects, such as neuroradiology, diagnostic imaging, etc. The levels of students in each study involves both undergraduates and graduates. The brand-new paradigm “flipped classroom” was adopted in all studies as intervention measure, while traditional didactic teaching mode or lecture-based learning (LBL) pedagogy was carried out as a contrast. The outcome indicators included theoretical scores, practical scores, and subjective evaluation.

**Table 1:**
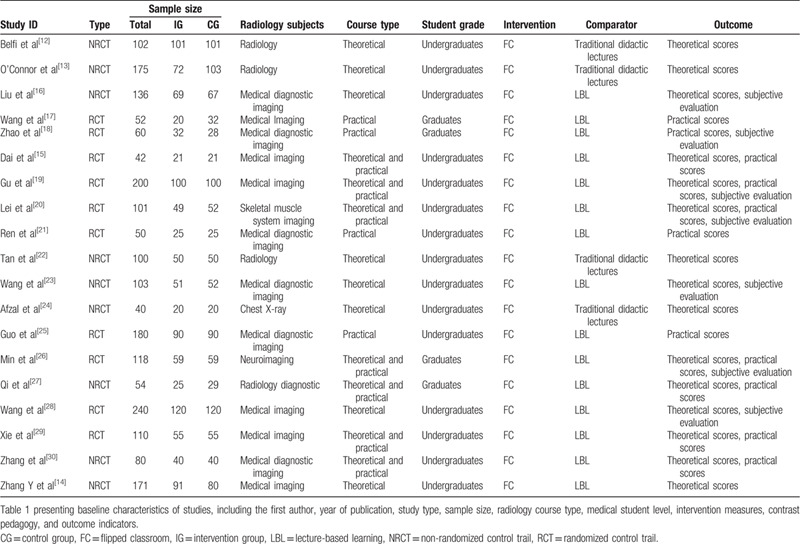
Characteristics of included studies.

### 4.3 Quality assessment

The methodological quality of each included study is presented with detailed scores in Table 2.[^12^,^13^,^14^,^15^,^16^,^17^,^18^,^19^,^20^,^21^,^22^,^23^,^24^,^25^,^26^,^27^,^28^,^29^,^30^] All these 19 studies were awarded a total score ranging from 5 to 11 points and meet the cut-off value of high-quality studies with respect to study design.

**Table 2:**
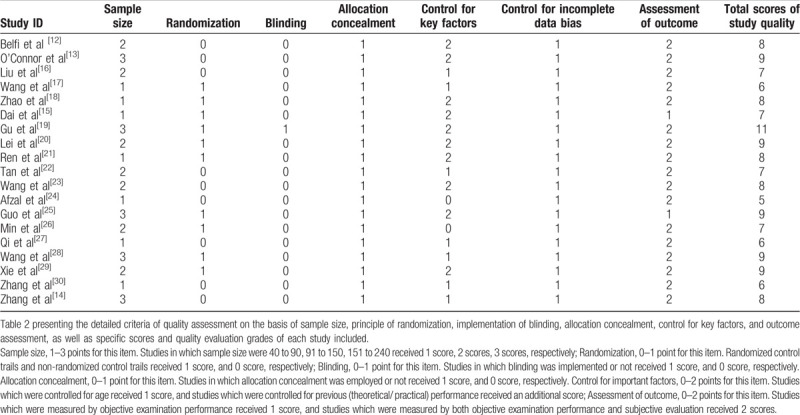
Methodological quality assessment of included studies.

## 5 Meta-analysis results

### 5.1 Theoretical examination performance

**Figure 2:**
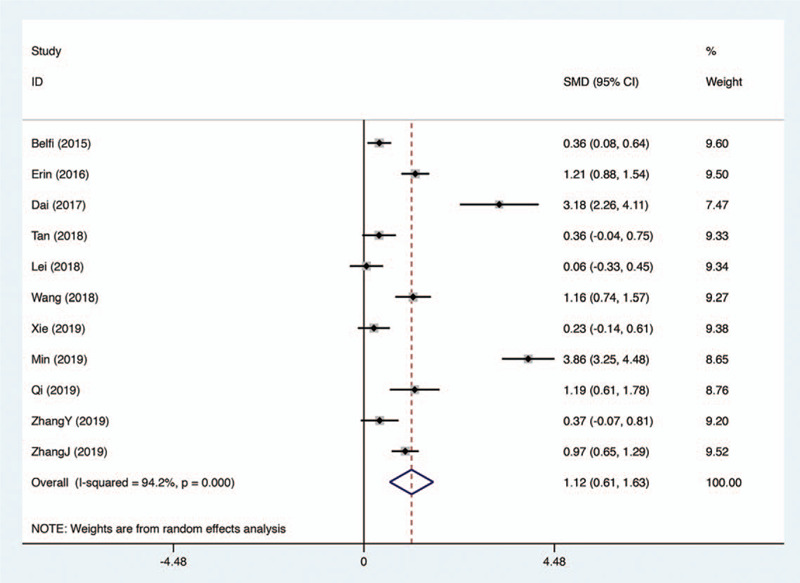
Forest plot for the effectiveness of flipped classroom vs traditional lectures on theoretical examination performance.

A total of 11 studies provided comparison of the effect on medical students’ theoretical examination performance between flipped classroom and traditional teaching-learning approach. Significant heterogeneity was seen across studies (*I* ^2^ = 94.2%), so that random effect model was applied. As shown in Figure 2, the meta-analysis result revealed that the evolution of flipped classroom significantly promoted the theoretical scores of medical students in radiology courses compared with passive learning (SMD 1.12, 95% CI 0.61–1.63, *P* < .001).

### 5.2 Practical examination performance

**Figure 3:**
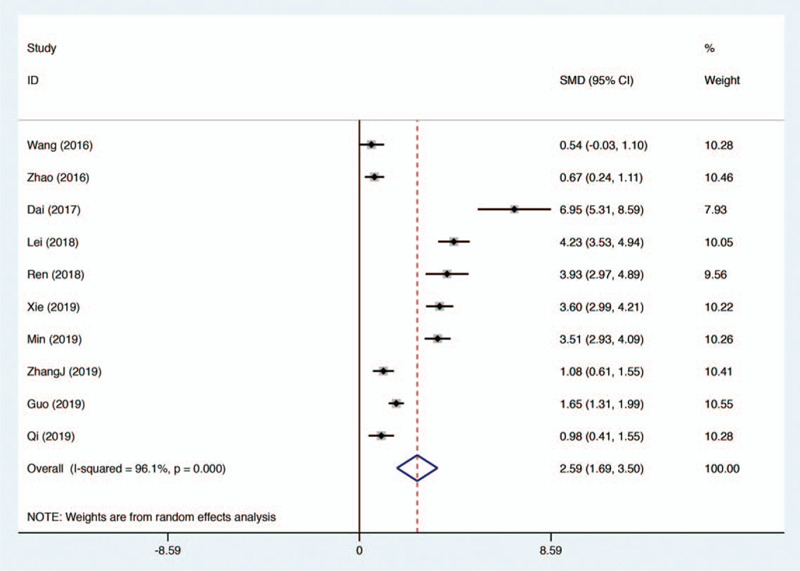
Forest plot for the effectiveness of flipped classroom vs traditional lectures on practical examination performance.

Altogether, 10 eligible studies were included to investigate the effectiveness of flipped classroom in the application of radiology education. Random effect model was used for evaluation, as significant heterogeneity was detected across all of the 10 studies (*I* ^2^ = 96.1%). As shown by the pooled result (Fig. 3), flipped classroom successfully improved the practical scores by introducing blending learning (SMD 2.59, 95% CI 1.69–3.59, *P* < .001).

### 5.3 Subjective evaluation results

**Figure 4:**
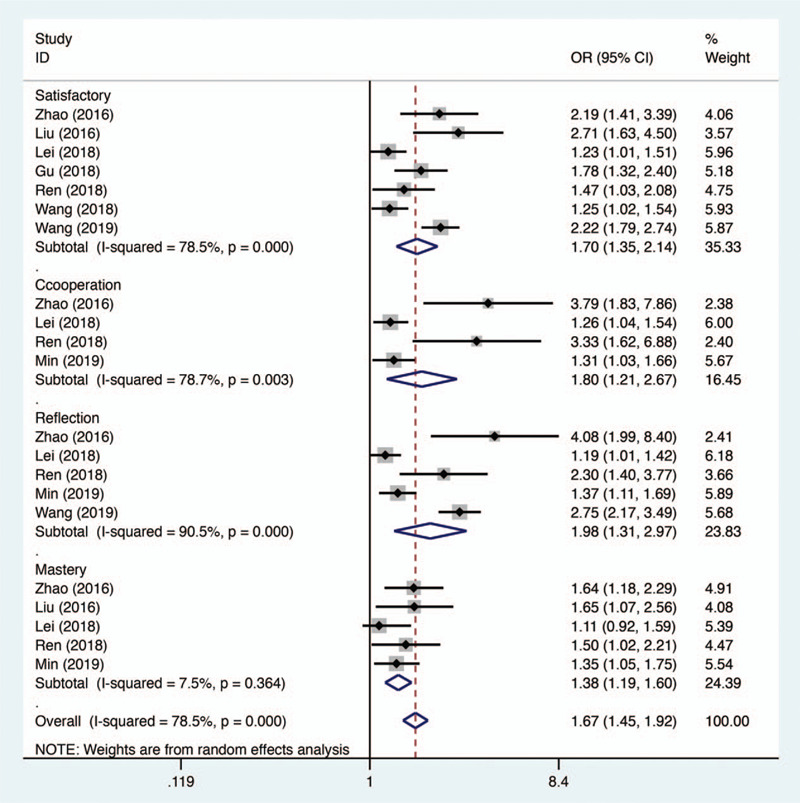
Forest plot for the effectiveness of flipped classroom vs traditional lectures on subjective evaluation indexes.

Four aspects, including course satisfaction, improvement of self-directed learning and reflection, cooperation ability, and consolidation of knowledge mastery were comprehensively analyzed to estimate the subjective cognition of medical students toward teaching methods transform (Fig. 4). Seven studies were included to evaluate students’ satisfaction with different pedagogical approaches. The meta-analysis results showed that compared with traditional instructor-centered lectures, the innovative active learning methods proposed by flipped classroom satisfied more students and gained higher appraise (OR 1.70, 95% CI 1.35–2.14, *P* < .001). Four studies provided the comparison of subjective assessment of cooperation ability improvement under the instruction of 2 different education strategies, and a significant advantage was shown in pooled results (OR 1.80, 95% CI 1.21–2.67, *P* = .004). To evaluate the effectiveness of flipped classroom in enhancing autonomous learning and reflection capacity, 5 studies were included and yielded a statistically positive response (OR 1.98, 95% CI 1.31–2.97, *P* = .001) demonstrating the superiority of flipped classroom over traditional mode. Another 5 studies were included to estimate the influence of teaching approaches on students’ subjective opinion to knowledge mastery. The meta-analysis results displayed that students agreed to the concept that this newly developed pedagogical method was conducive to the consolidation of knowledge (OR 1.38, 95% CI 1.19–1.60, *P* < .001).

### 5.4 Subgroup analysis

**Figure 5:**
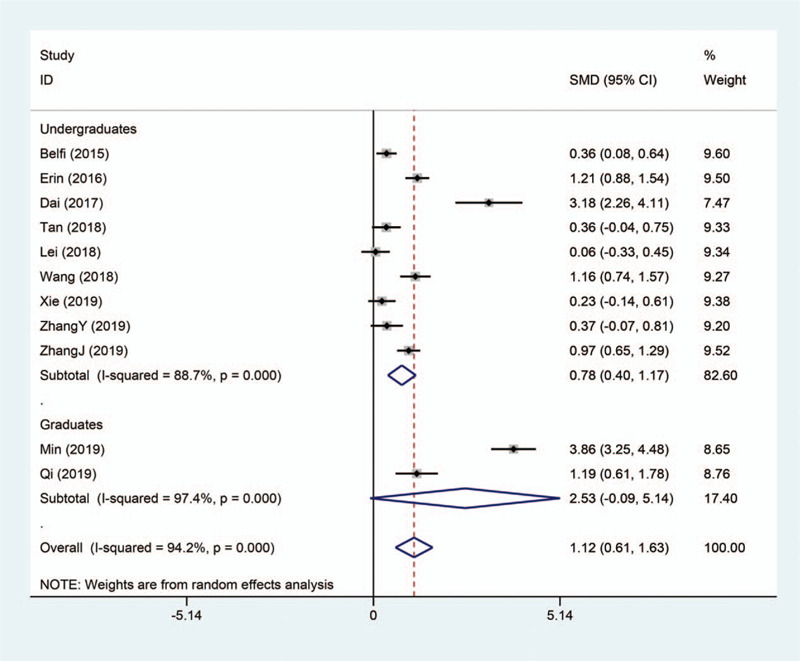
Subgroup analysis on theoretical examination performance.

**Figure 6:**
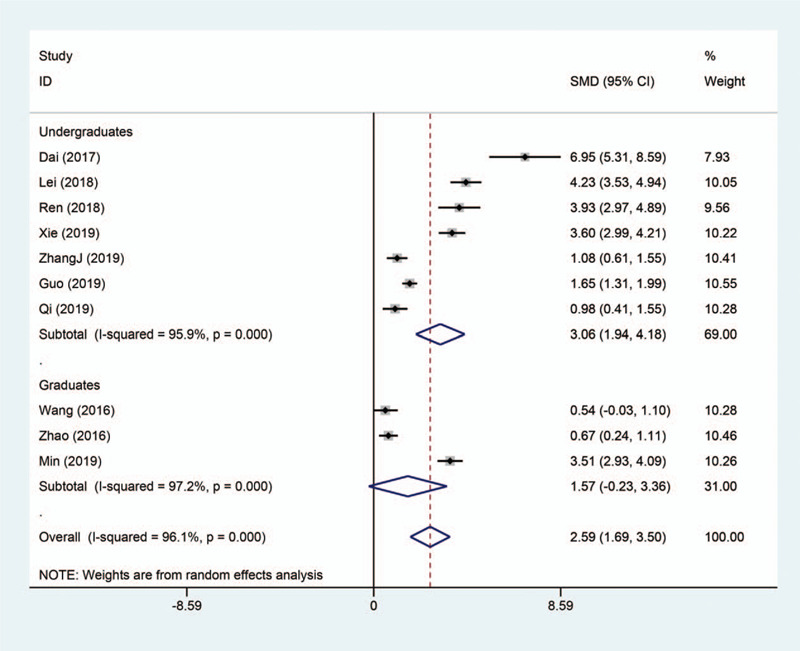
Subgroup analysis on practical examination performance.

We performed subgroup analyses based on different levels of medical students, including undergraduates and graduates. The results showed that there was still significant heterogeneity in different subgroups, which further illustrated that the study population was not the source of heterogeneity (Figs. 5 and 6).

### 5.5 Publication bias

**Figure 7:**
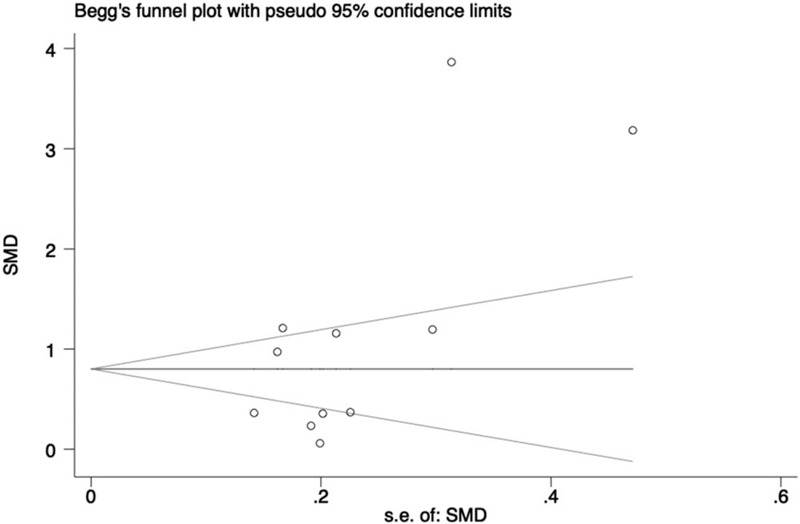
Funnel plot of theoretical examine scores with pseudo 95% confidence limits (Egger *P* = .044).

**Figure 8:**
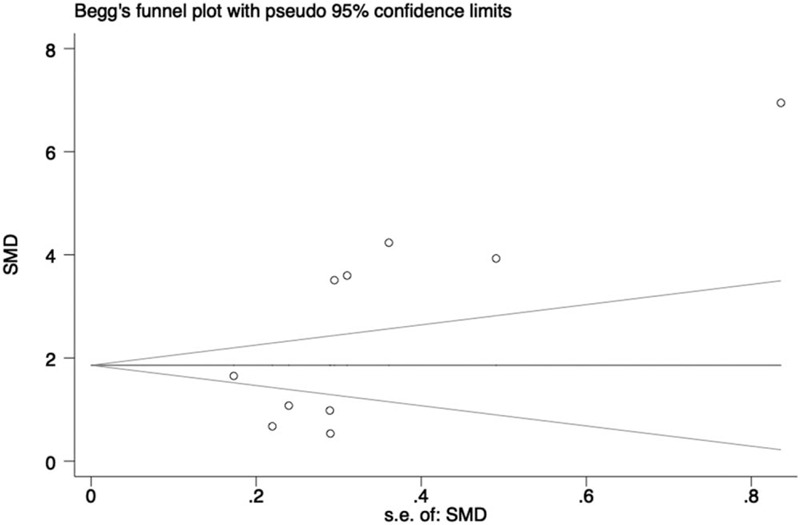
Funnel plot of practical examine scores with pseudo 95% confidence limits (Egger *P* = .038).

For the indicators with no less than 10 studies included, we conducted publication bias analysis. The Begg funnel plot asymmetry and corresponding Egger *P* value were displayed to estimate the publication bias. The existence of publication bias was indicated in both theoretical and practical examine scores with Egger *P* < .05 (Figs. 7 and 8). However, no evidence of publication bias was seen with the trim and fill method, which further supported the reliability of results.

## 6 Discussion

### 6.1 Summary

Overall, the results yielded in this meta-analysis referred that the subversive flipped classroom applied in radiology course has advantages over traditional lecture-based passive pedagogical method in promoting both theoretical and practical performance. Moreover, students’ preference was also attained in the process of flipping the classroom. To gain a detailed understanding of the unique strengths of this recently emerged learning-teaching model, explanations can be listed as follows. First, flipped classroom makes full use of the modern technique and platform to transform “cramming teaching” into “active learning,”^[11]^ which completely replace the situation of unidirectional transmission of knowledge from teacher and fundamentally stimulate students’ independent learning motivation in radiology.^[12]^ Second, the prerecorded videos enable students to arrange study plans at their own pace, without restriction of time and place. For the knowledge points poorly mastered, students can play the videos multiple times according to the actual situation, which not only caters to students’ satisfaction with teaching materials but also promotes students’ learning quality and efficiency practically.^[13]^ Third, the liberated in-class time provides more opportunities for problem solving and group discussion, making up for the disadvantages of short in chance to fulfill idea expression and peer interaction. Only then can teachers distinct the problems among students and explain them in a targeted way.^[14]^ After repeated thinking and deep understanding, medical students can form a profound memory of the content rather than learning by rote in the traditional class. Fourth, in view of the characteristics of radiology education, instructors can make the best of the class time to carry out case sharing and discussion on the basis of ensuring that students master basic knowledge through extracurricular self-centered learning. The in-depth analysis of medical records can significantly broaden students’ clinical thinking and help to narrow the gap between theory and practice.^[13]^ Medical students trained in the mode of flipped classroom are believed to adapt to the transformation from students to doctors much better.

However, there still exists some disputes on the reform of pedagogical approaches in radiology education. First, it is widely accepted that the traditional teacher-oriented lecture has been accustomed by a majority of students. Participants who prone to the traditional teaching mode hold the view that extracurricular preview can take up a lot of spare time and further add to their academic burden. The competitions and activities that originally took part in fail to continue due to the introduction of flipped classroom. Second, the extensive conduction of flipped classroom in radiology pedagogy is supposed to consume generous human, material, and financial resources.^[15]^ Third, the consensus of self-regulated learning can be different from person to person, and it is quite hard to implement supervisory control over students in the process of pre-class videos watching.^[15]^ Consequently, the disparity between students will be further widened. These findings thus indicated the fact that instructors who advocated the application of flipped classroom are obliged to convey the intention and value of the innovative curriculum model to all students in a detailed way and truly inspire the enthusiasm of self-oriented learning without the strict supervision from teachers. Meanwhile, reasonable control of videos’ duration and rigorous manage of the quality of online teaching materials can also do benefit to the conduction of flipped classroom.

### 6.2 Strengths and limitations

Radiology serves as a bridge course to link the basic medical knowledge and clinical practice. The rapid development of medical and science technique puts forward new requirements for the medical talents training. As a recently emerged pedagogical approach, the flipped classroom has been attempted in the education of radiology. To our knowledge, our meta-analysis is the first one to systematically evaluate the effectiveness of flipped classroom vs traditional lectures in the application of radiology courses, covering both subjective and objective assessments with the summary of scientific evidence.

However, several limitations also exist in our study still need to be addressed. First, the detailed course designs of the flipped classroom, including the form, content, and duration, were not specifically described in some included literature, of which the difference may influence the actual pedagogical effectiveness. Second, this meta-analysis mainly focused on the application effectiveness of flipped classroom in comparison with traditional instructor-led mode. However, other newly developed pedagogical approaches such as problem-based learning (PBL), and team-based learning (TBL), were not taken into consideration. To better illustrate the advantages of flipped classroom over other teaching-learning models, more comparative analysis with standard designs and consistent outcome assessment should be conducted in the future to gain significant reference value.

## 7 Conclusion

The result in our meta-analysis revealed that flipped classroom in radiology education possesses multiple strengths over traditional lectures in improving the overall performance and satisfaction of medical students. Under the premise of reasonably grasping the conduction rhythm and learning intensity, the pedagogical model of flipped classroom is well worth popularization in radiology courses to replace the traditional passive learning mode and prepare students for adapting clinical practice in the near future.

## Acknowledgments

The authors are sincerely appreciated to all the funding supports, as well as the editor and reviewers.

## Author contributions

Lingling Ge and Yuntian Chen collected, screened the literature and wrote the manuscript together. Jiaming Liu pointed out writing design and carefully revised the manuscript to control the overall quality. Chunyi Yan and Zhengwen Chen proposed meaningful comments on the manuscript. All authors read and approved the final manuscript.

**Conceptualization:** Lingling Ge.

**Funding acquisition:** Jiaming Liu.

**Writing – original draft:** Lingling Ge.

**Writing – review & editing:** Jiaming Liu.
